# Ecological Realism Accelerates Epigenetic Aging in Mice

**DOI:** 10.1111/acel.70098

**Published:** 2025-05-21

**Authors:** Matthew N. Zipple, Ivan Zhao, Daniel Chang Kuo, Sol Moe Lee, Michael J. Sheehan, Wanding Zhou

**Affiliations:** ^1^ Laboratory for Animal Social Evolution and Recognition, Department of Neurobiology and Behavior Cornell University Ithaca New York USA; ^2^ Center for Computational and Genomic Medicine The Children's Hospital of Philadelphia Philadelphia Pennsylvania USA; ^3^ Department of Pathology and Laboratory Medicine University of Pennsylvania Philadelphia Pennsylvania USA

**Keywords:** aging, DNA methylation, epigenetics, social interaction, wild environment

## Abstract

The aging of mammalian epigenomes fundamentally alters cellular functions, and such changes are the focus of many healthspan and lifespan studies. However, studies of this process typically use mouse models living under standardized laboratory conditions and neglect the impact of variation in social, physical, microbial, and other aspects of the living environment on age‐related changes. We examined differences in age‐associated methylation changes between traditionally laboratory‐reared mice from Jackson Laboratory and “rewilded” C57BL/6J mice, which lived in an outdoor field environment at Cornell University with enhanced ecological realism. Systematic analysis of age‐associated methylation dynamics in the liver indicates a genomic region‐conditioned, faster epigenetic aging rate in mice living in the field than those living in the lab, implicating perturbed 3D genome conformation and liver function. Altered epigenetic aging rates were more pronounced in sites that gain methylation with age, including sites enriched for transcription factor binding related to DNA repair. These observations underscore the overlooked role of the social and physical environment in epigenetic aging with implications for both basic and applied aging research.

## Graphical abstract

Living environment shapes mammalian aging, but laboratory conditions lack the ecological realism of natural social, physical, and microbial environments. Comparing traditionally laboratory‐reared and “re‐wilded” C57BL/6J mice, we reveal accelerated, genomic region‐dependent epigenetic aging in the liver under naturalistic field conditions, highlighting the overlooked impact of environmental factors on epigenetic aging.

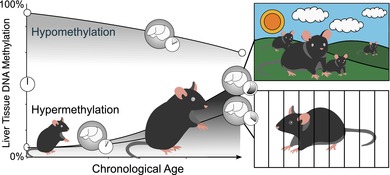

## 1 Introduction

Most biomedical research is conducted on model organisms living under standardized laboratory conditions (Rosenthal and Brown 2007). Unlike natural populations, model organisms living in lab settings have stable access to food and shelter, experience mild, near‐constant climatic conditions, and often have limited, static social experiences (Zipple et al. 2023). One consequence of keeping short‐lived animals in the laboratory is that median lifespans are dramatically extended, partially through reduced extrinsic mortality from predators and competition (Tidière et al. 2016). Laboratory conditions may also influence intrinsic cellular processes such that the magnitude of age‐related molecular changes differs between laboratory and field conditions, with downstream consequences for senescence.

Changes in DNA methylation are a molecular hallmark of aging (Jung and Pfeifer 2015; López‐Otín et al. 2023). Global methylation tends to decline with age, whereas methylation of specific CpGs, for example, at CpG islands, becomes more methylated with age (Zhou and Reizel 2025; Jones et al. 2015). These age‐associated methylation changes are often used as a biometric of human and nonhuman animal health, with individuals showing accelerated epigenetic aging being at increased risk of morbidity and mortality (Levine et al. 2016; Belsky et al. 2022; Simpson and Chandra 2021; Thompson et al. 2018; Horvath and Raj 2018; Horvath 2013). The aging epigenome also mechanistically contributes to other cellular and physiological changes linked to aging, such as stem cell functional deficit and malignancy risk (Jung and Pfeifer 2015).

The extent to which age‐associated epigenetic change is shaped by environmental context and whether the changes observed under laboratory context accurately generalize to more ecologically realistic conditions remain open questions. Recently, a study of free‐living wild house mice trapped in Wales identified increased age‐associated rates of change in DNA methylation patterns in a handful of loci compared to inbred laboratory controls (Hanski et al. 2024), suggesting that free‐living conditions may increase rates of epigenetic change in mice. However, wild house mice are genetically distinct from inbred laboratory strains, and rates of epigenetic aging are known to be influenced by genetic variation, making causal conclusions about environmental influences challenging (McCartney et al. 2021; Lu et al. 2018).

Here, we leverage the genetic uniformity of isogenic laboratory mice and the ecological realism provided by naturalistic field enclosures to (1) assess the extent to which the laboratory environment influences age‐associated methylation changes genome‐wide and (2) identify a set of CpG sites in mice that are strongly affected by differences in environmental experiences.

## 2 Results

To address these questions, we compared rates of epigenetic aging between mice reared in a standard laboratory environment at Jackson Laboratory and mice whom we bred in the laboratory at Cornell University before releasing them as infants into a semi‐natural outdoor environment (hereafter “rewilded” mice).

**FIGURE 1:**
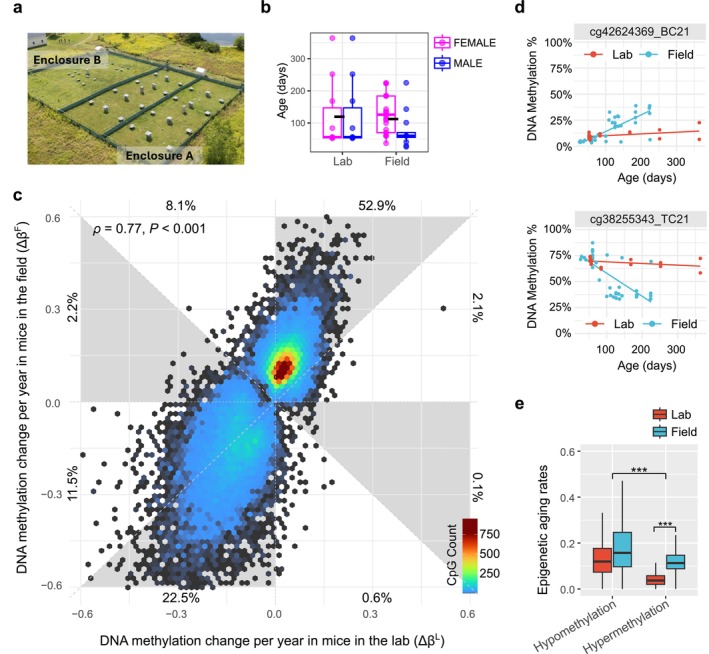
Comparison of age‐related epigenetic changes in laboratory versus field environments. (a) An aerial view of the two enclosures where the field cohort lived. (b) Field and laboratory cohorts, broken down by environment, age, and sex (color). (c) Comparing epigenetic aging rates between laboratory (X‐axis) and field mice (Y‐axis) among those sites that show age‐associated hyper‐ or hypomethylation in mouse liver tissues. The rates represent the estimated change in methylation level per year at a particular CpG site in each environment for sites that show significant age associations in at least one environment. The percentages of sites falling into the eight evenly spaced angular octants are labeled next to the axes. (d) Representative CpGs with faster epigenetic aging rates in the field than in the laboratory (upper panel: Age‐associated hypermethylation; lower panel: Age‐associated hypomethylation). (e) Boxplots comparing the distributions of absolute annual rates of epigenetic change at sites that become hypo‐ and hypermethylated in each environment.

To generate rewilded mice, we released infants of the common laboratory mouse (strain C57BL/6J) into a controlled outdoor field environment (Figures 1a and S1a), which offers dramatically increased social and physical ecological realism compared to standardized laboratory conditions (Zipple et al. 2023). This approach allows us to retain lab models' manipulability, reproducibility, and molecular tools while allowing for dynamic and complex physical and social experiences.

We collected 41 liver tissue samples from rewilded mice 26–225 days of age (Figure 1b) that had lived in the field enclosure since they were 13–15 days old (±1 day, i.e., infancy). We then compared the liver methylomes of these rewilded samples with those collected from 20 C57BL/6J mice of similar age ranges that lived their whole lives in a standard laboratory colony (Figure 1b). As validation, we also investigated a larger population of rewilded mouse liver tissue samples (*N* = 73), including the 41 described above and supplemental mouse groups that we placed into our field enclosures as adults (Figure S1a–c).

To measure the environmental impact on rates of change in methylation, we modeled cytosine methylation levels (percentage cytosine modified) at ~275 K CpG sites (from ~285 K after filtering missing values) for mice reared in each environment (laboratory or field). We modeled percent methylation as a function of age, sex, and environment to calculate epigenetic aging rate estimates at each CpG site (i.e., the rate of DNA methylation change with respect to age, calculated as the slope estimate between methylation level and chronological age). Separately, we also explicitly modeled the interaction between age and living environment at each CpG site to test whether the rate of epigenetic change at each individual site was significantly influenced by the environment.

Mice that developed in the laboratory versus the field are moderately congruent in the annual rate of methylation change across CpG sites (Figure 1c). Specifically, among CpGs that show age‐associated methylation change (*q* < 0.01 in at least one environment), there is a strong relationship between rates of epigenetic aging in the two environments (Spearman's rho = 0.77, *p*‐value < 0.001, *N* = 30,497). This strong correlation indicates site‐specific intrinsic propensities of the DNA methylation drift (Figure 1c). This pattern remains robust if we include supplemental samples from mice moved from the laboratory to the field later in adulthood (Figure S1d). Thus, although epigenetic aging rates are sensitive to the environment (see below), the mechanisms involved in this aging are largely unaffected by environmental differences. Representative CpGs with similar rates of methylation aging, faster aging in the field than in the laboratory, and vice versa, are shown in Figures 1d and S1e–f.

To further quantify this consistent relationship between epigenetic aging rates in the two environments, we categorized age‐associated methylation changes into eight octants, depending on the direction and magnitude of change in each environment (Figure 1c). A total of 55.0% (52.9% + 2.1%) and 34.0% (11.5% + 22.5%) of the age‐associated methylations are bi‐cohort joint hypermethylations and hypomethylations, respectively. In contrast, 10.3% (8.1% + 2.2%) of CpGs show hypermethylation in the field but hypomethylation in the laboratory. Under 1% of CpGs show hypomethylation in the field but hypermethylation in the laboratory. Age‐associated hypomethylations tend to have larger age‐related coefficients than hypermethylations (Figure 1e), consistent with a global decline in methylation levels with age.

Age‐associated changes in methylation are notably faster in mice reared in the field than in the laboratory (Figures 1c and S1d). Of age‐associated joint hypermethylations, 96% (i.e., 52.9% in 55.0%) have faster epigenetic aging rate estimates in the field. Among sites that show significant (*q* < 0.01) increases in methylation in both environments, the average rate of epigenetic change is approximately 94% faster in animals living in the field (95% CI = 91%–98% faster, zero‐intercept linear model, Figure S1g). A similar but dampened effect is seen in age‐related hypomethylations. A total of 66% of the joint hypomethylations (i.e., 22.5% in 34.0%) have faster aging rate estimates in the field (Figure 1c). Among sites that show significant decreases in methylation in both environments, the average rate of epigenetic change is 28% faster in the field (95% CI = 26%–29%, zero‐intercept model, Figure S1g). This intriguing difference between hyper‐ and hypomethylation suggests that the epigenetic aging of the two groups of CpG sites may be of different cellular mechanisms and variably affected by environmental differences.

A complementary analytical approach is to determine how many CpG sites, among those with significantly different rates of age‐related methylation change between the two environments, exhibit faster changes in the field. Among hypermethylation sites that are significantly age‐associated in both environments (*q* < 0.01) and that display a significant age‐by‐environment interaction (*q* < 0.05), all display faster rates in the field than in the laboratory (370/370 sites, 100%). A similar but slightly dampened pattern is seen among joint hypomethylations (498/529 sites, 94%).

In addition to the 41 animals reared from infancy in the field, we also collected liver samples from additional field‐exposed animals introduced to the enclosures in adulthood (*N* = 32). The above environmental effects on epigenetic aging were qualitatively similar when these animals were included in our analyses (*N* = 73 for field mice, Figure S1d).

**FIGURE 2:**
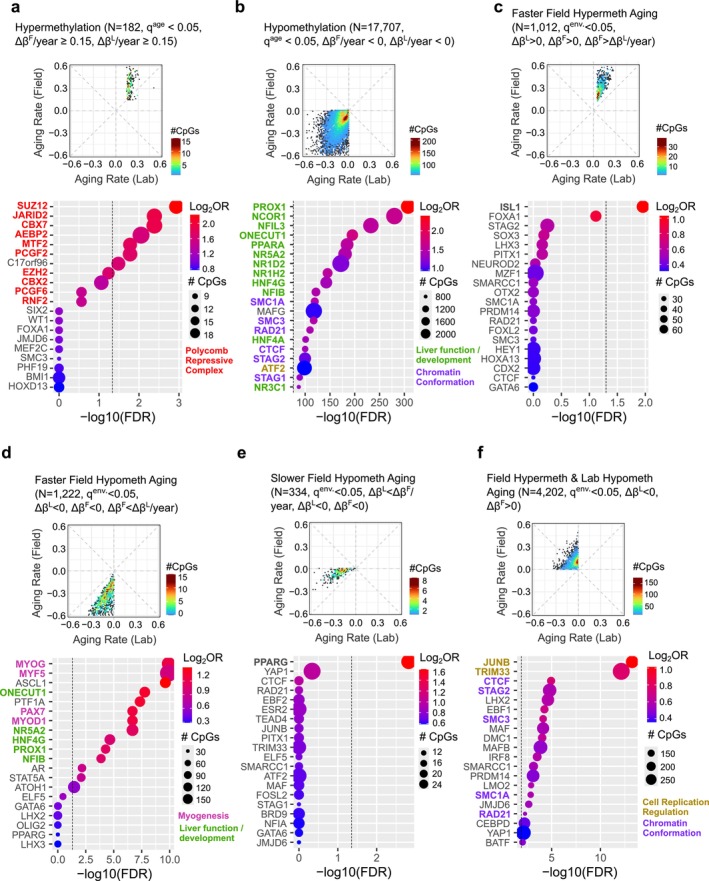
Functional enrichment analysis of age‐associated methylations with congruent and discrepant rates between mice living in the laboratory and the field. Transcription factor binding sites enriched for (a) CpG sites that gain methylation with age in both laboratory and field‐living mice; (b) sites that lose methylation with age in both laboratory and field‐living mice; (c) sites that gain methylation faster with age in the field than laboratory mice; (d) sites that lose methylation faster in the field than the laboratory‐living mice; (e) sites that lose methylation faster in the lab than field mice; (f) sites that gain methylation with age in the field‐living mice but lose methylation with age in the laboratory‐living mice. Transcription factor binding peaks were obtained from mouse ENCODE databases and overlapped with the mouse methylation array probes. CpG sites with age‐associated *q*‐values smaller than 0.05 were used as queries.

We performed functional enrichment analyses to further explore the differences in epigenetic aging rates observed in different environments. Broadly, sites that displayed congruent patterns of hyper‐ and hypomethylation under both environments reflect previously reported patterns of age‐associated methylation change. Those sites that display substantial hypermethylation with age in both environments primarily localize to bivalent promoters, poised enhancers, and binding sites of the Polycomb repressive complexes (e.g., SUZ12, JARID2, CBX7, AEBP2, MTF2, and PCGF2, see Figure 2a), consistent with prior reports of their association with replicative epimutation (Zhou and Reizel 2025) and role in stem cell differentiation (Schlesinger et al. 2007; Teschendorff et al. 2010). Sites that became substantially hypomethylated with age in both environments localize to the binding sites of transcription factors key to hepatocyte development and function (e.g., PROX1, NCOR1, ONECUT1, NR5A2, and NFIB, see Figures 2b and S2b), suggesting the role of these hypomethylation events in age‐associated liver maturation. Joint hypomethylations are also linked to the binding of cohesin complex proteins (e.g., SMC3, SMC1A, RAD21, and STAG2) and CTCF. These scaffold proteins involved in 3D nuclear conformation reflect previously reported age‐associated chromatin architectural changes (Zhou et al. 2022). Sex predicted methylation status, but sex‐associated methylations were exclusively enriched on sex chromosomes and at DMC1 binding sites in both the laboratory and rewilded cohorts (Figure S2a).

Sites that gain methylation faster with age in the field exhibit discordant patterns between mice that entered the field enclosure as infants versus adults. When only including mice that enter the field in infancy, sites with faster methylation gains are enriched for ISL1 binding. ISL1 is a multi‐tissue transcription factor that regulates genes, including insulin (Zhang et al. 2009). The hypermethylation of ISL1 binding sites may be linked to metabolic adaptation to the field environment, as liver expression of this transcription factor inhibits lipogenesis and promotes lipolysis in coordination with KDM6B and SNAI1 (Zhao et al. 2022). Interestingly, when including supplemental mice that entered the field during adulthood, sites that gain methylation faster in the field are enriched for sites bound by two DNA damage repair genes, TDG and RAD23B (Figure S2c), suggesting a link to age‐related DNA damage (Schumacher et al. 2021), epigenetic silencing (Sriraman et al. 2020), and repair. Increased DNA damage and its repair is a molecular hallmark of aging (Li et al. 2024; López‐Otín et al. 2023), and our results indicate that the rate of epigenetic change linked to this process depends on both environmental conditions and the developmental stage of environmental exposure. Within this supplemented analysis, sites that gain methylation are also enriched for DNA binding of proteins involved in chromatin conformation (SMC1A, CTCF, SMC3, and STAG1, Figure S2c,d), suggesting age‐associated 3D genome conformation impacts shaped by both gains and losses of methylation. Few sites gained methylation more rapidly in the laboratory than in the field, without any significant enrichment of binding sites associated with these changes.

Sites that lose methylation more rapidly in the field are moderately associated with transcription factors conventionally involved in the development of both hepatocytes (ONECUT1, NR5A4, and HNF4G) and non‐hepatocytes, such as muscle cells (MYOG, MYF5, PAX7, and MYOD1) and neurons (ASCL1) (Figures 2d and S2e). Whether the impact of these proteins on DNA methylation readouts is due to tissue composition change or altered hepatocyte expression warrants further investigation. Assessing differences in rates of methylation change in other tissue types represents a rich opportunity to understand tissue and system‐specific environmental impacts on the aging process. Sites that lost methylation more rapidly under laboratory conditions did not show a strong signal in the enrichment analysis (Figure 2e), with the only significant binding enrichment observed being PPARG. This gene also regulates lipid storage in the liver (Wang et al. 2020).

Next, we examined sites exhibiting divergent directions of change in epigenetic aging patterns between the laboratory and the field. Such CpGs were enriched at the binding of JUNB and TRIM33. Both proteins have been postulated as tumor suppressors and are involved in TGF‐beta signaling and chromatin regulation (Xi et al. 2011; Pérez‐Benavente et al. 2022, Figure 2f). While both field and laboratory‐reared mice already exhibited common methylation loss at cohesin complex and CTCF binding sites (Figure 2b), mice in the field uniquely gained additional methylation at a distinct subset of CpGs bound by these same complexes (Figure 2f). This observation aligns with the understanding that CTCF binding is highly responsive to DNA methylation, contingent on genomic and chromatin context (Héberlé and Bardet 2019). Our findings suggest that 3D chromatin conformation changes play a role in epigenetic aging and contribute to the differential rates of epigenetic change between the laboratory and the field. Unlike sites that display hypomethylation with age in both environments, no strong involvement of liver function and development is observed in these sites with divergent directions of age‐associated changes in methylation (Figure 2f).

Finally, we asked if the observed disparity in rates of epigenetic change is reflected in epigenetic clock measurements. Epigenetic clocks are predictive models for chronological and phenotypic age based on methylation levels (Meer et al. 2018; Thompson et al. 2018; Stubbs et al. 2017; Petkovich et al. 2017; Wang et al. 2017) or other molecular markers (Simpson and Chandra 2021; Jylhävä et al. 2017). Although not the focus of our study, we calculated epigenetic ages from two epigenetic clocks (Zhou et al. 2022) to test whether the observed global accelerations of age‐related changes in methylation were reflected in this more selective set of CpG sites. We applied a published epigenetic clock of 347 CpG features (Zhou et al. 2022) and a new epigenetic clock of 248 CpG features (Methods) to predict the age of each mouse tissue sample (Figure S2f). Both clocks were trained on multiple tissues from laboratory‐reared animals and tended to overestimate chronological age in both cohorts of liver samples. However, the overestimations are greater in the field cohort than in the laboratory cohort (median residual = 2.25 and 2.5 months in the field cohort, 1.4 and 1.8 months in the laboratory cohort, *p* = 0.003 and 0.01, Figure S2g). Despite clocks using a small fraction of CpG features in the genome, the higher prediction residuals confirm the above observation of differential epigenetic aging in the field versus the laboratory. Yet, relying only on the single quantitative output of such a clock conceals the variation in the magnitude of the environmental effects described above, which depend on whether sites become hyper‐ versus hypo‐methylated with age.

## 3 Discussion

Our results lead us to two conclusions. First, the rate of epigenetic changes in the most used biomedical model organism is highly dependent on environmental context, with laboratory‐reared animals showing a global bias toward slower rates of epigenetic aging compared to field‐reared animals. Second, this more rapid aging of the epigenome is particularly pronounced in sites that gain methylation with age, which are enriched for genes associated with insulin regulation, DNA damage repair, and CTCF and cohesin binding (Figures 2c, S2c and 1f). From the current data, it remains unclear if rewilded mice also show accelerated senescent phenotypes, including early onset disease development and behavioral declines, compared to those in the laboratory.

Our data also suggest that environmental impacts on epigenetic aging rates likely depend on the life stage and duration of exposure to different environments. While we currently lack the density of sampling to quantify exposure effects explicitly, epigenetic aging rate differences and CpG sites displaying such differences depending on the age of exposure were apparent in our data (Figures S1 and S2). This age‐dependent environmental impact suggests that age‐related DNA methylation changes follow a nonlinear pattern like other aging biomarkers (Shen et al. 2024). The present study provides a blueprint and motivation for future work identifying when animals are most susceptible to environmental impacts and where these life‐stage sensitivities lie in the epigenome.

Our data hold some possible insights into the mechanisms by which animals may display accelerated epigenetic aging in the field compared to the laboratory. From an environmental perspective, animals in the field are exposed to a wide range of different environmental challenges and opportunities, including (1) social competition and potential resource scarcity in males, (2) homeostatic challenges resulting from dynamic weather experiences, (3) social instability when animals die or are born, and (4) reproductive effort in the form of mating and territorial defense in males and pregnancy and reproduction in females. Each of these environmental experiences—which are faced to an extent by all natural populations of vertebrates, including humans—may have contributed to short‐term or chronic physiological stress, with downstream impacts on epigenetic aging rates. Our enrichment analysis provides some support for this interpretation, as particularly rapid methylation was observed at sites modifying the binding of transcription factors involved in tissue function (Figure 2b) and metabolism (Figure 2c), potentially also implicating tissue composition change (Figure 2d) and DNA repair (Figure S2c). Many of these factors differ in their intensity between the sexes, and future examinations of sex‐specific age acceleration at particular CpG sites or in particular tissues will be valuable.

Here, we have focused our analysis on the liver, which represents a proxy for overall metabolic output and also comes into substantial contact with environmental substances via detoxification. This work will benefit from future expanded analyses to include multiple tissues that come into relatively high contact (e.g., colon, skin, and lungs) or low contact (e.g., muscle) with the environment. Similarly, identifying particular cell types that are especially sensitive to environmental factors using single‐cell methods and characterizing the transcriptional and cellular mechanisms by which differences in these environmental experiences impact animals' physiology and aging trajectories is a rich opportunity for future work. And as we characterize these differences across additional tissue and age ranges, we will be able to synthesize our results with existing datasets that have used the same array we have used here (e.g., Razzoli et al. 2023).

One limitation of our study is that the laboratory‐reared tissues that we measured were obtained from Jackson Laboratory, while rewilded animals were bred at Cornell University from parents that originated at the Jackson Laboratory. Though measures of the same strain of inbred mouse are generally consistent across laboratories and time, laboratory‐specific differences in both behavior and physiology have been reported, which are occasionally large enough to alter qualitative conclusions (Crabbe et al. 1999; Mandillo et al. 2008; Kafkafi et al. 2018; Jaric et al. 2022; Nigri et al. 2022). Given the consistent global bias in age‐related changes in methylation that we observe and the magnitude of the environmental difference between the field enclosures and a standard laboratory environment, it is unlikely that this difference in laboratory sources for our control tissues explains our results. However, we are unable to rule this difference out as an explanatory factor.

Mice hold a central position in aging research. Our results highlight the need to consider the environmental context of model organisms when studying aging‐related phenotypes. Although therapeutics targeted at physiological and behavioral decline are usually tested on mice living in standard conditions, these mice are physiologically distinct from the same genotype, experiencing more ecologically realistic conditions. Both mice and humans face fluctuating physical and social environments in natural populations. Our results demonstrate the importance of this variation in the molecular mechanisms of aging and highlight the importance of incorporating as much of this variation as possible into the lives of model organisms (Shemesh et al. 2024; Lee et al. 2022; Karamihalev et al. 2020).

## 4 Methods

### 4.1 Field Enclosures and Study Subjects

Field study subjects were reared and processed at Cornell University. Data from laboratory study subjects (see below) were obtained from control animals aged at the Jackson Laboratory.

We generated our field study subjects by breeding 9‐week‐old male and female C57BL/6J mice that we purchased from Jackson Laboratory (Bar Harbor, ME) and mated in our laboratory at Cornell University. Upon female pregnancy, males were removed from breeding cages to prevent re‐insemination following parturition. When pups were 8–10 days of age, we anesthetized litters and their mothers using brief isoflurane exposure (< 5 min) and injected either 1 (pups) or 2 (mothers) RFID tags (Biomark Mini HPT10) subcutaneously with a 20‐gauge needle. The RFID tag is a permanent identification method to identify known‐aged individuals following recapture from our field enclosures.

Our field subjects considered in the main text are all derived from this group of animals initially released as pups into one of our field enclosures (Enclosure A), in which we are able to monitor individual animals' behavior. These animals' social and spatial behavioral data during development were analyzed and published in a separate project (Zipple et al. 2025). The enclosures at Cornell University's Liddell Field Station have been described in detail previously (Vogt et al. 2024). Briefly, from infancy through adulthood, the subjects in the main text lived in an enclosure 15 m × 38 m in size, approximately 9000 times the area of a typical mouse cage from infancy through ~60 days of age. In this enclosure, we setup 16 plastic tubs (31‐gal storage totes, Rubbermaid, USA), placed into four neighborhoods of four resource zones (Figure S1b). Each tub (hereafter “resource zones”) contained ad libitum food access and a nestbox that provided insulation and shelter from adverse weather conditions. We equipped each zone with a single joint entrance/exit made from a 6‐in.‐long PVC pipe (2 in. in diameter). These resources and the single entrance made the resource zones highly valuable and defendable, mimicking commensal mice's foraging landscape.

After field subjects reached approximately 62 days of age, those that had not yet been killed for tissue collection (*n* = 26) were transferred to another one of our field enclosures (Enclosure B, not equipped with behavioral monitoring technology), approximately 20 m away, to continue their lives (Enclosure A, and its tracking technology was needed for another project). Enclosure B is approximately 1.5 times the size of Enclosure A, and we setup resource zones in Enclosure B in the same pattern as Enclosure A. These animals were then opportunistically captured over the next 6 months to generate our dataset of known‐age tissue samples (Figure S1a).

#### 4.1.1 Animals Included in Supplemental Analyses Only

We took tissue samples opportunistically from animals other than those included in the main text (Figure S1a,b). These supplemental animals fall into three groups. First, we obtained tissue samples from some of the mothers of the animals in the main text (*n* = 10, hereafter Supplemental Group 1), who we also placed outside in our enclosure alongside these main study subjects (who were dependent infants at the time of initial exposure). Second, when we transferred field subjects from Enclosure A to Enclosure B, we also introduced an additional cohort of animals into Enclosure B to supplement our data collection efforts. This cohort of animals was made up of (1) individuals of the same age as the infant‐exposed field subjects but who had spent their lives under standard laboratory conditions (hereafter Supplemental Group 2; *n* = 12) and (2) the mothers of the individuals in Supplemental Group 2 (hereafter Supplemental Group 3, *n* = 10).

### 4.2 Rewilded Mice Liver Methylome Profiling

Individuals were recaptured from field enclosures either by hand‐trapping or overnight capture using Sherman traps. For all sample collection, animals were humanely killed via cervical dislocation followed by decapitation. We then collected one liver lobe and flash‐froze the tissue on dry ice. Tissues were then stored at −80°C until DNA extraction. We extracted DNA from 25 mg of liver tissue, following the DNeasy Blood and Tissue Kit for DNA Extraction protocol (Qiagen N.V.). We stored the extracted DNA at −80°C until methylome profiling.

According to the manufacturer's protocol, bisulfite conversion of 500 ng input liver DNA per sample was performed using EpiTect Bisulfite Kits (Qiagen, 59104). The Infinium Mouse Methylation BeadChip assays were conducted at the Center for Applied Genomics Genotyping Core of the Children's Hospital of Philadelphia.

### 4.3 Laboratory Mouse Liver Methylome Profiling

C57BL/6J mouse liver tissue was directly acquired from the Jackson Laboratory. Extraction of genomic DNA from mouse liver tissues follows previous work (Kaur et al. 2023). The whole liver tissue was homogenized using a tissue homogenizer (OMNI, TH115) in 500 μL of lysis buffer containing 10 mM Tris pH 8.0 (VWR, 97062‐674), 300 mM NaCl (VWR, 10128‐484), 0.5% SDS (Invitrogen, 15553027), and 5 mM EDTA (VWR, 10128‐442). After adding 15 μL of Proteinase K (NEB, P8107S), the solution was incubated at 55°C overnight. Subsequently, 100 μL of the solution was combined with an additional 400 μL of lysis buffer and incubated at 55°C for 2 h. The 500 μL of the solution was placed into a 5PRIME Phase Lock Gel tube (Quanta bio, 10847‐802) pre‐centrifuged for 1 min. 500 μL of phenol/chloroform/isoamyl alcohol (Sigma‐Aldrich 77617) was added to the phase Lock Gel tube. The aqueous phase solution was transferred to a new 1.5 mL centrifuge tube (Eppendorf, 05414203). Five hundred microliters of 100% isopropanol (MilliporeSigma, EM1.09634.1011), GlycoBlue (Invitrogen, AM9515), and Ammonium acetate solution 7.5 M (Sigma‐Aldrich, A2706) were added to the tube. The solution was vortexed and incubated at −20°C for 30 min up to O/N. Samples were centrifuged at 16,000 g for 30 min at 4°C and washed twice by adding 1 mL 70% EtOH (MilliporeSigma, EM1.00983.1011). After the last wash and removal of 70% EtOH, the samples were air‐dried for 10 min and resuspended in 200 μL of the Tris buffer pH 8.0 (VWR, 97062‐674) and incubated at 55°C for 10 min. DNAs that were not completely dissolved were incubated at 4°C overnight. If the dissolved DNA did not exhibit a transparent color or the DNA quantity was inadequate, an additional bead purification step was carried out. Briefly, 2× volume of AMPure XP (Beckman Coulter, A63881) was added to the DNAs, mixed thoroughly, briefly spun down, incubated the mixture for 5 min at room temperature, placed on a magnet stand, washed two times with 500 μL of freshly made 80% EtOH, and allowed to dry for 3–5 min. The final elution was performed using 100–200 μL autoclaved ultrapure water. DNA amount was measured using the Qubit 4 Fluorometer (Invitrogen) with the dsDNA HS Assay Kit (Invitrogen, Q33231).

DNA bisulfite conversion was performed using the EZ DNA Methylation Kit (Zymo Research, D5001) or EZ‐96 DNA MethylationTM MagPrep (Zymo, D5040). Samples with bisulfite converted by the EZ DNA Methylation kit were performed according to the manufacturer's instructions with the specified Illumina Infinium Methylation Assay modifications. Samples that bisulfite converted by EZ‐96 DNA MethylationTM MagPrep (Zymo, D5040) were performed using the same process as the above. The Infinium Mouse Methylation BeadChip assays were conducted at the Center for Applied Genomics Genotyping Core of the Children's Hospital of Philadelphia.

### 4.4 Public Laboratory Mouse Liver Methylome Dataset

We also included 10 C57BL/6J lab mouse liver samples from a prior study (GSE184410; see Table S1 for sample accessions). IDAT files were downloaded and processed using the openSesame workflow with default parameters.

### 4.5 Data Preprocessing and Analysis

All IDAT files were processed using the openSesame workflow with default parameters (Zhou et al. 2018). The data quality is assessed using the sesameQC pipelines. All samples have over 90% probes passing the signal detection threshold (pOOBAH detection *p*‐value < 0.05).

We produced two sets of estimated rates of age‐related change in methylation status using the DML function from the SeSAMe package (Zhou et al. 2018), one for our field cohort and one for our laboratory cohort. We built a third model that explicitly modeled this interaction to establish significant age‐by‐environment interactions. To assess a bias in the number of sites that showed a significant interaction between age and environment, we counted the number of significant interactions in each direction (i.e., rates in the field are faster than laboratory or vice versa) for CpG sites that significantly increased or decreased in methylation status over time in both cohorts.

To quantitatively assess the relationship between age‐related rates of change in methylation in each environment, we built a no‐intercept linear model (i.e., anchored to the point (0,0)) using estimated rates of change in each environment for sites where the age‐associated *q* value in each environment was < 0.01 (Figure S1g). These represent the subset of CpG sites in which we have the most confidence that methylation status changes with age. We derived an estimate and uncertainty value from this model for (1) sites that lose methylation in the laboratory with age and (2) sites that gain methylation in the laboratory with age.

### 4.6 CpG Probe‐Wise Enrichment Analysis

Enrichment testing in mouse ENCODE transcription factor bindings and other CpG databases for all probe sets in this work was performed using the *testEnrichment* function from the *knowYourCG* R package (version 1.0.0). The mouse Infinium BeadChip's cg probes are used as the background probe universe (Zhou et al. 2022). As the mouse array preferentially designed enhancer and promoter CpGs (Zhou et al. 2022), we used a higher threshold at 0.15 in testing methylation gains. Without thresholding, the gains showed no statistically significant term. We used a similar −0.15 threshold for hypomethylation, and the result is shown in Figure S2b.

### 4.7 Epigenetic Clock Analysis

We tested a previously published epigenetic clock that uses 347 features (Zhou et al. 2022). For validation, we developed another clock using a different set of 248 CpG methylation features. We derived the mouse epigenetic clock and biological age estimates using previously published data (Zhou et al. 2022). When constructing this clock, we collected 706 public mouse tissue methylomes (Table S1) and constructed an epigenetic clock for the MM285 array using an elastic net framework. The elastic‐net regularized linear model was built using glmnet. To select the most predictive CpGs, we set alpha to 0.5 and lambda to 0.1633, selected using the cv.glmnet function, which automatically optimizes the mean absolute error of the model using 10‐fold cross‐validation. Only autosomal probes were used as model features. This procedure leads to an epigenetic clock of 248 CpG probes with an estimated mean absolute error of 1.2 months.

## Author Contributions

This project was first conceptualized by M.N.Z. and M.J.S., and field mouse liver samples were provided by M.N.Z., D.C.K., and M.J.S. and profiled by S.M.L. M.N.Z., I.Z., M.J.S., and W.Z. participated in the data analysis. M.N.Z., M.J.S., and W.Z. oversaw the project.

## Conflicts of Interest

The authors declare no conflicts of interest.

## Supporting information


Figure S1.

Table S1.

## Data Availability

The generated mouse methylome profiles (*N* = 93) are available in the Gene Expression Omnibus with accession GSE269932. Other public laboratory mouse liver methylomes (*N* = 7) and additional samples for epigenetic clock construction can be found in GSE184410 (sample accessions listed in Table S1). Informatics for mouse methylation data preprocessing and functional analysis is available in the R/Bioconductor package *SeSAMe* (version 3.22+): https://bioconductor.org/packages/release/bioc/html/sesame.html. The epigenetic clock is available at https://github.com/zhou‐lab/CytoMethIC_models/blob/main/models/Age_MM285_20230101.rds.
