# Supplementary material for: Ecological Realism Accelerates Epigenetic Aging in Mice
Source: Aging Cell. 2025 May 21;24(6):e70098. doi: 10.1111/acel.70098 (PMC12151873; doi:10.1111/acel.70098)
Supplement: Supplementary file 1 — Figure S1. [file ACEL-24-e70098-s002.pdf]

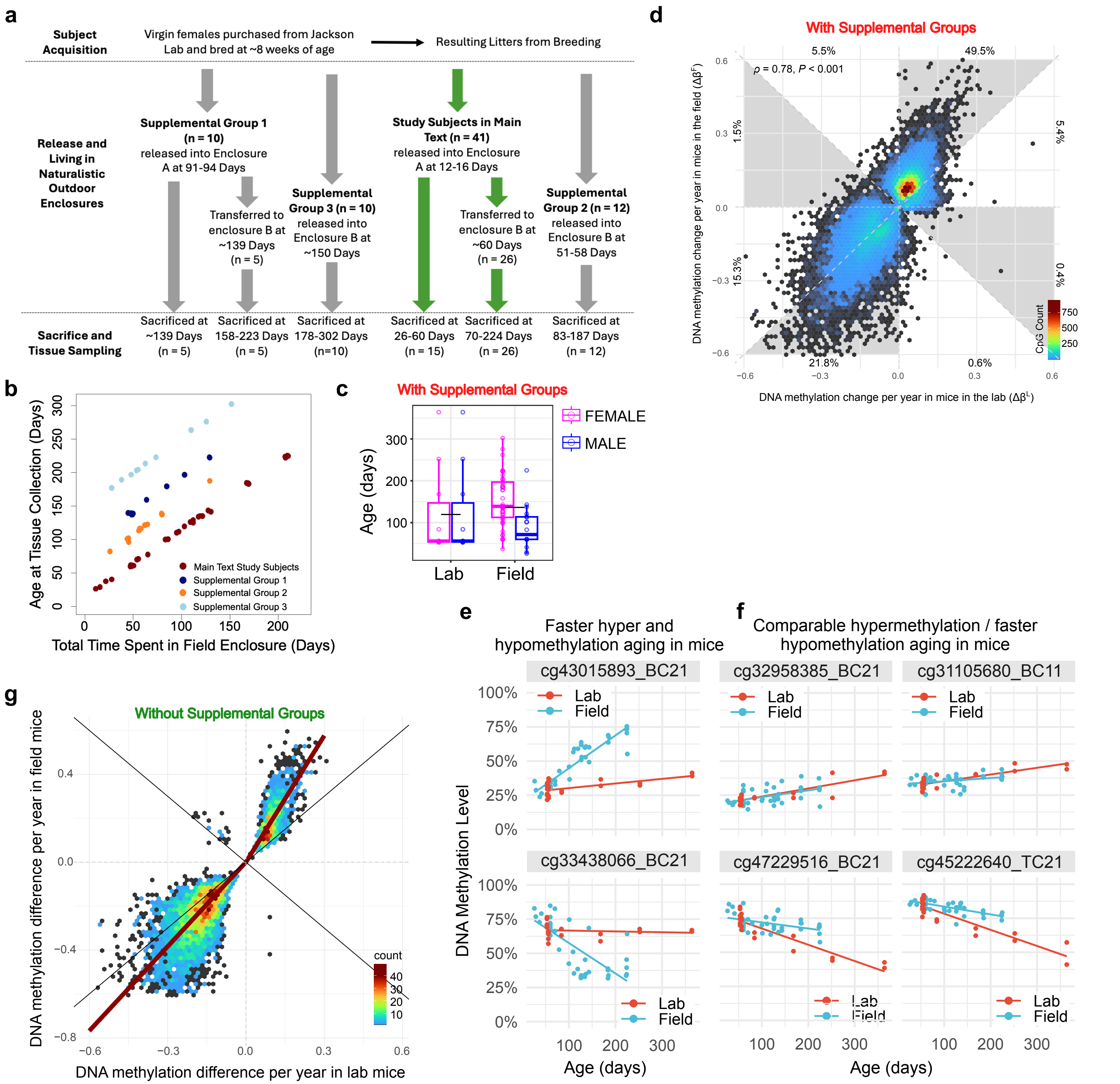

Supplemental Figure S1: (a) Schematic of the life experiences of the subjects in this study. Animals considered in the main text (n = 41) spent nearly all their lives in our field enclosures under semi-natural conditions. Supplemental Groups 1-3 spent some portion of their lives in our field enclosures and are considered in the supplement. (b) Scatter plot of individuals' ages at the time of tissue collection (y-axis) versus the time spent in the enclosures (x-axis). Points are jittered to prevent overlap. N=73 tissue samples are plotted. (c) Age distribution of the larger cohort, including both the samples brought to the field during infancy (main text) but also the supplemental groups. (d) The comparison of methylation change per year comparing lab and field-reared mice, when supplemental groups are included. (e-f) Representative CpGs that display differential epigenetic aging, comparing age in days (X-axis) and methylation level (Y-axis) in faster aging in field mice (e) and lab mice (f). Supplemental groups are not included. Top row shows comparable or faster hypermethylation and bottom row shows faster hypomethylation. (g) The comparison of methylation change per year contrasting lab and field mice, showing significant, joint hyper and hypomethylation probes in zero-intercept models. Both methylation loss and methylation gain is globally accelerated in the field environment (null expectation is the y = x line).

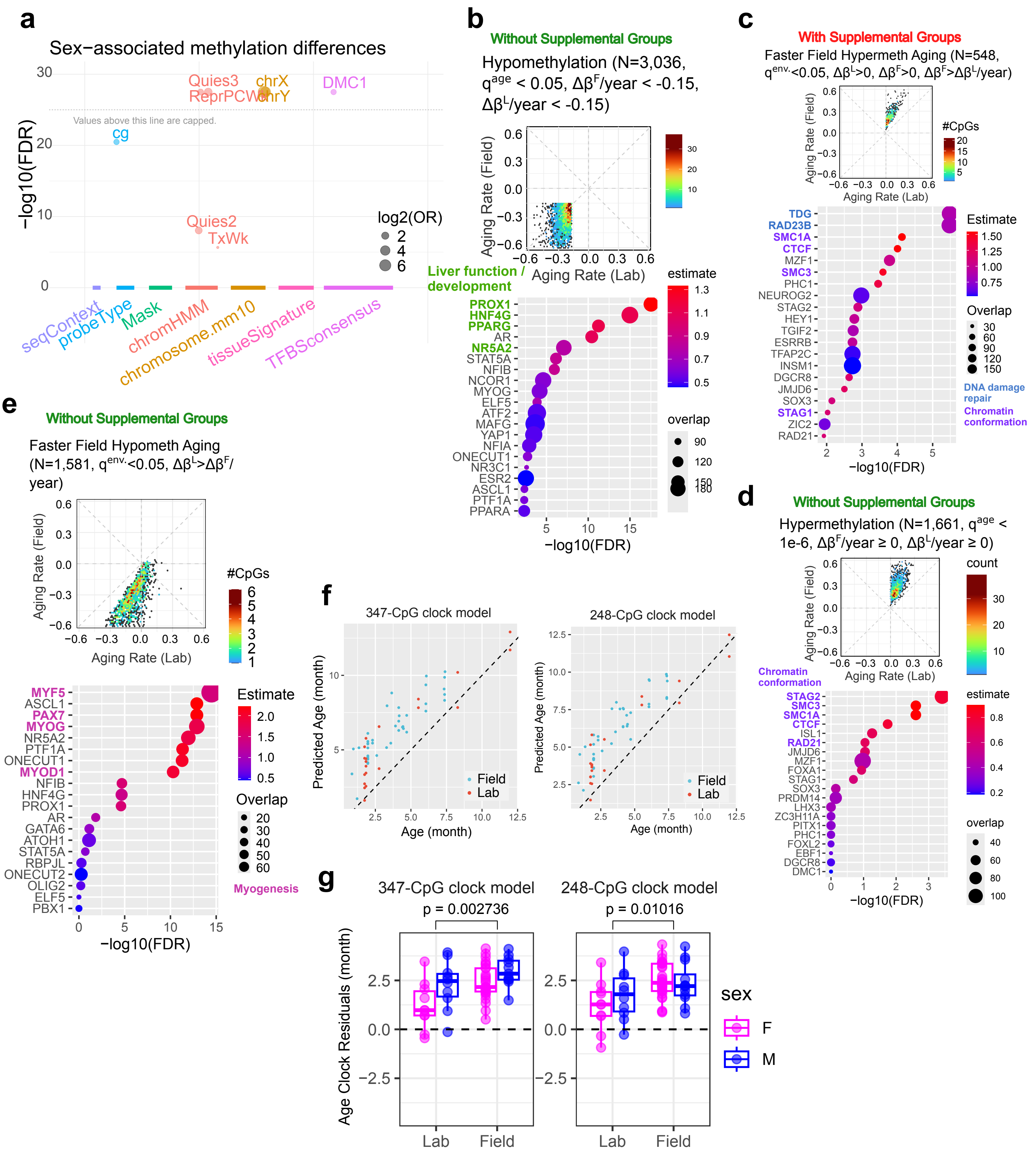

Supplemental Figure S2: (a) Sex-associated methylation differences shows enrichment on the sex chromosomes, as expected. (b-e) Functional enrichment analysis of age-associated methylations that lose in both the field and the lab-living mice, thresholding greater effect size (-0.15) (b), gain faster in the field than lab mice (c-d), lose faster in the field than lab mice (e); This is the same as Figure 2d except that no directionality is restricted, hence including a small number of sites that gain methylations in the lab-living mice. (f-g) Epigenetic clock reveals accelerated biological aging in field mice using 248 and 347 CpG methylation features. (f) Scatter plot comparing clock predictions (both 347-CpG model and 248-CpG model) in lab and field mice. (g) Box plot comparing epigenetic clock prediction residuals in lab and field mouse cohorts in two models stratified by sex. p-value were based on Wilcoxon rank sum test merging both sexes.
